# Different patterns and characteristics of Talar injuries at two main orthopedic trauma centers in Shiraz, south of Iran

**DOI:** 10.1186/s12891-021-04486-0

**Published:** 2021-07-06

**Authors:** Amir Reza Vosoughi, Reza Fereidooni, Saeedreza Shirzadi, Seyed Alireza Zomorodian, Amir Human Hoveidaei

**Affiliations:** 1 Orthopedic Foot and Ankle Surgeon, Department of Orthopedic Surgery, School of Medicine, Shiraz University of Medical Sciences, Shiraz, Iran; 2 Student Research Committee, Shiraz University of Medical Sciences, Shiraz, Iran; 3 Students’ Scientific Research Center, Tehran University of Medical Sciences, Tehran, Iran

**Keywords:** Accidents, Ankle, Dislocation, Epidemiology, Fracture, Talus

## Abstract

**Background:**

Categorizing different injury patterns of the talus, describing demographic data, mechanisms of injury and associated fractures are important issues in orthopedic trauma surgeries. Injuries of the talus require careful attention with appropriate treatment approaches in order to reduce possible complications.

**Methods:**

In a cross-sectional study, the demographic characteristics, mechanism of injury, fracture type, and associated fractures were compiled from all patients’ files and operation notes with diagnosis of talar injuries from January 2014 to December 2019.

**Results:**

Among 367 patients, 317 (86.4%) males and 50 (13.6%) females with mean age of 31.8 ± 11.6 years were identified. There were three (0.8%) patients with bilateral talar fractures. The most common mechanism of injury was motor vehicle accident (MVA) (46.1%), followed by falls (43.3%), direct trauma (6.2%) and sport injuries (4.4%). About half of the patients injured in MVAs were motorcyclists. Isolated talar body fractures (21.9%) were more common than isolated talar neck (19.2%) or combined body & neck fractures (14.6%). Isolated lateral process fracture is the most frequent fractured process of the talus (14.3%). Hawkin type IIA (39.2%) was the most common type of talar neck, followed by Hawkin type III (22.3%), type I (21.5%), type IIB (14.6%) and type IV (2.3%). Medial malleolus, fibula and calcaneus were the most common associated fractures, respectively.

**Conclusions:**

The population that is most affected by talar injury are active young men who are involved in motor vehicle accidents, especially motorcycle crashes, with fracture of body and/or neck of talus being the most common type.

## Background

Fractures and dislocations of the talus are rare injuries with potentially severe complications. It accounts for less than 1% of all fractures and 2–6% of foot and ankle fractures. The incidence of talus fracture is estimated to be 3.2/100,000 per year [1–5]. Injuries of the talus require careful attention with appropriate treatment approaches and accurate post-treatment follow-up visits in order to reduce possible complications such as wound infection, joint stiffness, muscle atrophies, osteonecrosis, degenerative arthrosis, malunion and nonunion [6]. Notably, each of these complications could bring a great long-term disability for these patients with a significant reduction in quality of life [7, 8].

The most common reported mechanism for talar injuries is high-energy trauma including motor vehicle accidents (MVA) and falls. Several associated bony and soft tissue injuries have been reported in patients with talar fracture [1, 9–11].

Epidemiological studies can determine the causes, demographic data, different patterns and characteristics of an injury. These researches can have a positive effect in better organization of trauma services and help the health system provider to reduce the cost burden by preventing the etiologic factors [12]. By means of literature review, there are a few published studies on the epidemiology and demographic data of foot and ankle injuries [1, 3, 4, 9, 13–20]. To the best of our knowledge, there are few studies focused on all epidemiological aspects of talar fracture. Two studies were performed in Brazil with 24 and 36 cases of talar fracture [1, 9]. Also, Anandasivam et al., in 2019, explained demographic data and associated injuries of talar fractures without determining different patterns and classifications by evaluating 25,615 patients according to the National Trauma Data Bank of the United States [13]. Based on the nature of epidemiological studies, these findings may vary in different countries and societies [21].

This study aimed to categorize different injury patterns of the talus and describe demographic data, mechanisms of injury and associated fractures for each type by reviewing all talar injuries treated at the two main orthopedic trauma teaching hospitals of Shiraz, the biggest city in the south of Iran, during a 6-year period.

## Methods

After approving the study by the ethic committee of Shiraz University of Medical Sciences (IR.SUMS.REC.1398.161), in a cross-sectional study, all consecutive patients with the diagnosis of talus fracture and/or dislocation, from January 2014 to December 2019, in a level I trauma center (Emtiaz Hospital) and the main orthopedic trauma teaching hospital (Chamran Hospital) in Shiraz, South of Iran were enrolled and reviewed.

Demographic characteristics and job of the patients, mechanism of injury, length of hospital stay, the interval between admission and surgery, treatment approaches and associated fractures were compiled from the patients’ files and operation notes. For completing the missing data, we asked the patients by phone.

Radiological images and computed tomography (CT) scans of the patients were reviewed by the senior author (ARV) in order to determine the anatomical regions of the injury. Differentiation between body and neck fractures was based on the inferior part of the fracture line. If it was anterior to the lateral process, it was classified as the talar neck fracture [22]. Hawkins classification for talar neck fractures was designated [23].

Statistical analysis was performed using the statistical package for the social sciences version 16.0 for Windows (SPSS Inc. Chicago, IL, USA). Descriptive data were expressed as frequency and mean.

## Results

**Fig. 1:**
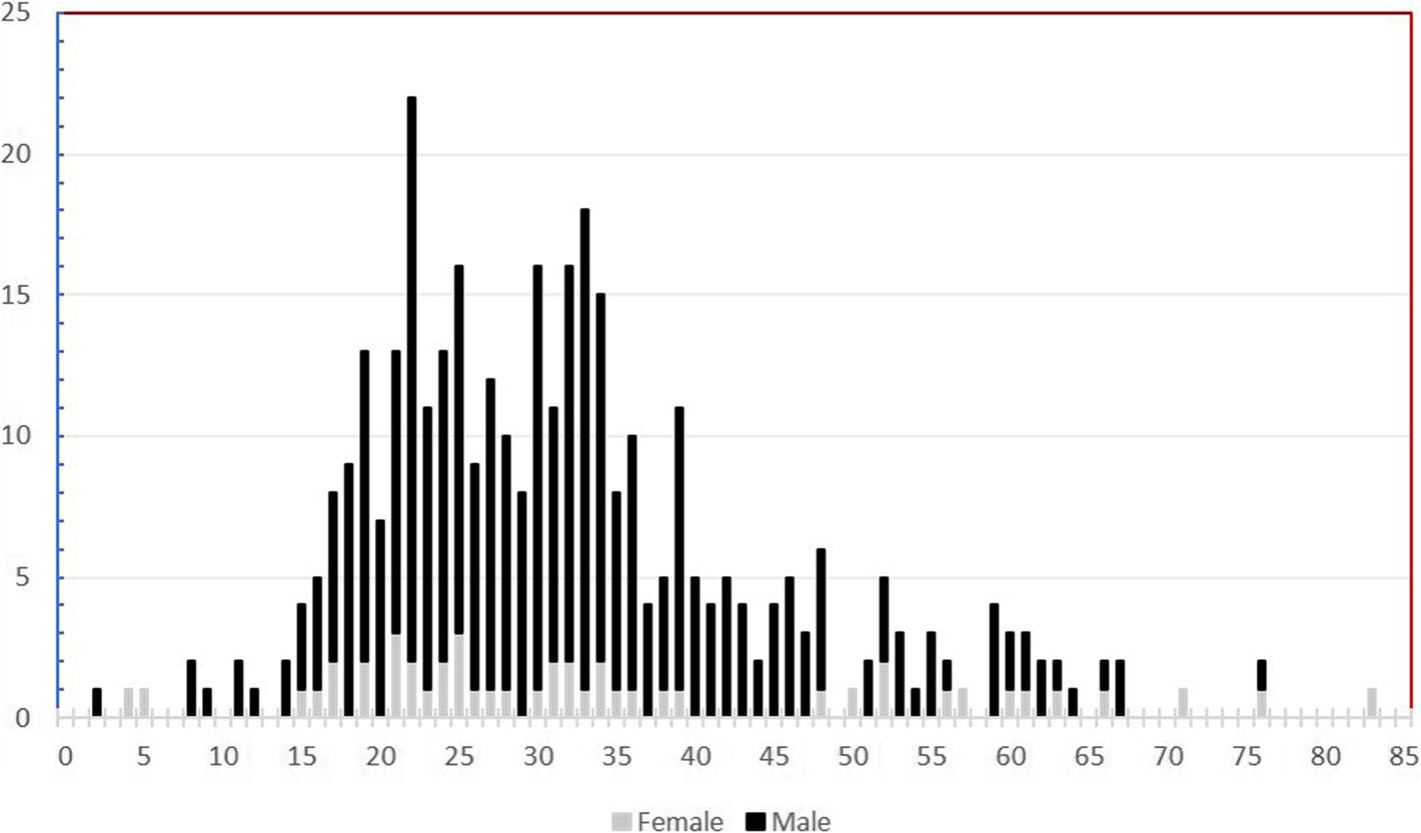
The distribution of patients by age and gender

**Fig. 2:**
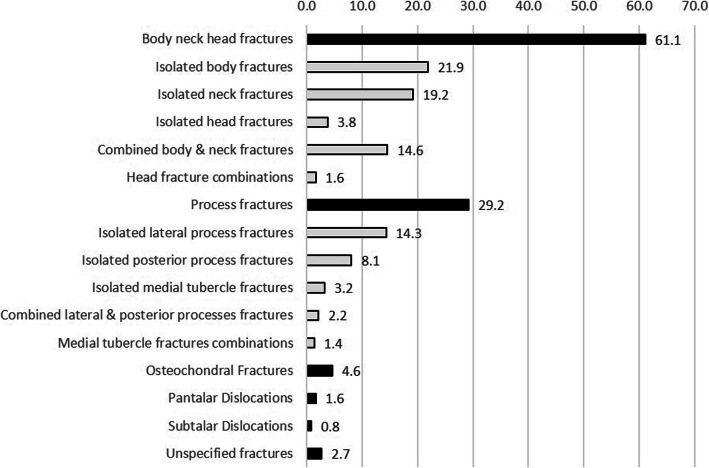
Frequency of talar injuries (%)

Totally, 367 patients with 370 talar fractures and/or dislocation were identified. There were 317 (86.4%) males and 50 (13.6%) females with mean age of 31.8 ± 11.6 years. The main affected group was young men between 18 to 45 years of age. Twenty-nine patients (7.9%) were younger than 18 and 2.2% were older than 65 years (Fig. 1). Right talus was injured more frequently than the left (56.5% vs 43.5%). There were three patients (0.8%) with bilateral talar fractures. Twenty-six cases (7.0%) had an open talar injury. As shown in Fig. 2, talar body neck head fractures were the most common injured area followed by talar process fractures.

Based on the available occupation of 179 cases, there were 31.8% workers, 29.7% freelancers or those with self-employed jobs, 12.8% students, 6.1% engineers, 5.6% housewives, 5.0% unemployed cases, 2.8% farmers, 2.8% soldiers, 1.7% drivers and 1.7% retired individuals.

**Fig. 3:**
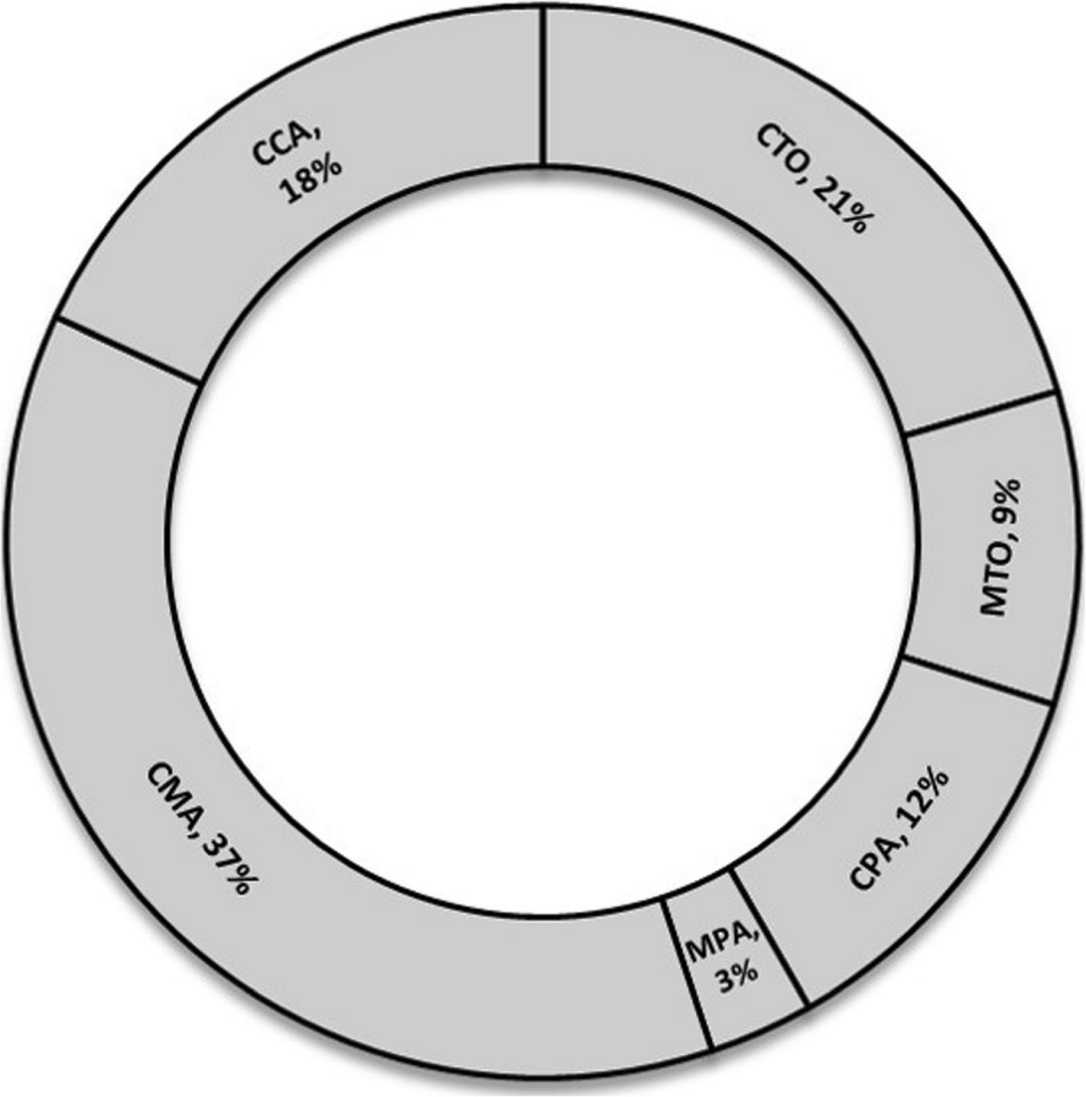
Different kinds of motor vehicle accidents

The mechanism of injury was available for 321 (87.5%) patients (324 talar injuries) (Table 1). As illustrated in Fig. 3, car to motorcycle accident was the most frequent kind of MVA, accounting for 127 cases. The mean height of fall was 3.2 ± 2.4 m. The most common mechanism of injury among the elderly population (> 65 years) was falling down (75%), while it was MVA among young patients.

**Table 1:** Mechanisms of injury distribution by talar injury patterns (324 injuries)

	Body neck head fractures					Process fractures					Osteochondral fractures	Pantalar Dislocations	Subtalar Dislocations	Unspecified fractures	Total
	Isolated body fractures	Isolated neck fractures	Isolated head fractures	Combined body & neck fractures	Head fracture combinations	Isolated lateral process fractures	Isolated posterior process fractures	Isolated medial tubercle fractures	Combined lateral & posterior processes fractures	Medial tubercle fracture combinations					
**MVA**	**34**	**32**	3	21	3	**22**	**14**	3	**5**	0	6	**4**	**2**	1	150
**Fall**	31	20	**5**	**26**	2	20	9	**5**	2	**5**	**7**	0	0	**7**	139
**Direct Trauma**	3	9	3	1	0	2	1	1	1	0	0	0	0	0	21
**Sport Injury**	2	3	2	1	0	4	1	0	0	0	1	0	0	0	14

*MVA* motor vehicle accident

Bold numbers are indicative of the most common injury mechanism for each kind of talus fracture and/or dislocation

Most of the cases (67.8%) were surgically managed. The mean length of hospital stay was 7.29 (range, 1–59) days. The mean time between the trauma and the surgery was 3.82 (range, 0–28 days.

**Fig. 4:**
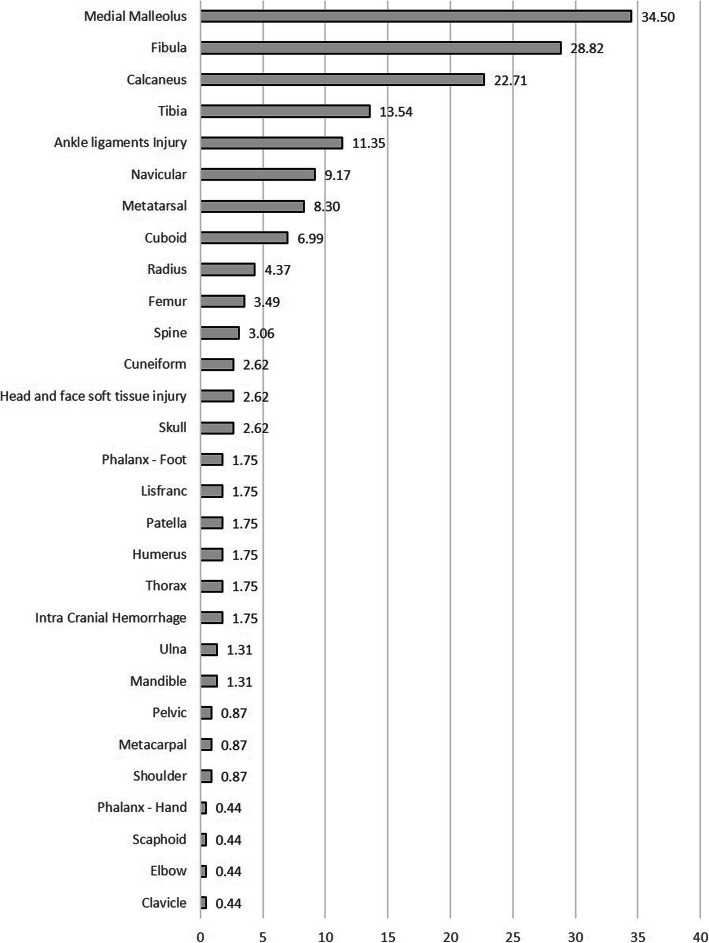
Associated injury frequency (% among 229 cases)

Overall, 229 (62.4%) patients with talar fracture and/or dislocation had associated injuries. The associated injuries occurred in the ipsilateral side in 204 (89.1%), contralateral side in 11 (4.8%) and bilateral in 14 (6.1%) cases. Medial malleolus, lateral malleolus (fibula) and calcaneus were the most common associated fractures, respectively (Fig. 4).

Among surgically-treated patients with associated ankle ligament injuries, deltoid rupture was the most frequent ligament injury followed by anterior talofibular, ankle syndesmosis and calcaneofibular ligaments. Also, there were 3 cases with rupture of the flexor retinaculum and 10 cases of superior peroneal retinaculum rupture or avulsion-fracture with dislocated peroneal tendons. Notably, some cases had multiple ligament injuries of the ankle joint.

### Body neck head fractures

The frequency and demographic data of all kinds of body neck head fractures of the talus are demonstrated in Tables 2 and 3.

1. Isolated body fractures

**Table 2:** Frequency of different types of talar fractures

Type	Frequency (%)
**Body head neck fractures**	226 (61.1%)
Isolated body fracture	81 (21.9%)
Isolated neck fracture	71 (19.2%)
Hawkin type I	25 (6.8%)
Hawkin type IIA	33 (8.9%)
Hawkin type IIB	13 (3.5%)
Isolated head fracture	14 (3.8%)
Combined body & neck fracture	54 (14.6%)
Hawkin type I	1 (0.3%)
Hawkin type IIA	15 (4.1%)
Hawkin type IIB	6 (1.6%)
Hawkin type III	29 (7.8%)
Hawkin type IV	3 (0.8%)
Head fracture combinations	6 (1.6%)
Head & neck	3 (0.8%)
Head & body	1 (0.3%)
Head & neck & body	2 (0.5%)
**Process fractures**	108 (29.2%)
Isolated lateral process	53 (14.3%)
Isolated posterior process	30 (8.1%)
Isolated medial tubercle	12 (3.2%)
Lateral & posterior processes	8 (2.2%)
Lateral process & medial tubercle	4 (1.1%)
Posterior process & medial tubercle	1 (0.3%)

**Table 3:** Distribution of body neck head fractures by age and gender

Age & gender	−18		18–39		40–64		65+		Male/ Female ratio	Total
	♀	♂	♀	♂	♀	♂	♀	♂		226
Isolated body	1	5	7	47	1	18	1	1	7	81 (35.8%)
Isolated neck	5	3	8	50	0	5	0	0	4.5	71 (31.4%)
Isolated head	0	0	1	9	0	3	0	1	13	14 (6.2%)
Combined body & neck	0	7	6	36	0	4	0	1	8	54 (23.9%)
Head combinations	0	1	1	4	0	0	0	0	5	6 (2.7%)

There were 81 cases of isolated body fractures, including 30 (37.0%) nondisplaced fractures, 48 (59.3%) displaced fractures without joint subluxation and 3 (3.7%) displaced body fractures with subluxation of the subtalar joint. There were four cases (4.9%) of open fractures.

2) Isolated neck fractures

Among 71 patients with isolated talar neck fractures (Table 4), five cases (7.0%) had open fractures, four cases of Hawkins type IIA and one Hawkins type I.

3) Isolated head fractures

**Table 4:** Frequency of neck fractures regarding Hawkins classification

Hawkins classification	Isolated neck	Combined body & neck	Combined neck & head	Combined body & neck & head	Total
I	25	1	2	0	28 (21.5%)
IIA	33	15	1	2	**51 (39.2%)**
IIB	13	6	0	0	19 (14.6%)
III	0	29	0	0	29 (22.3%)
IV	0	3	0	0	3 (2.3%)
Total	71 (54.6%)	54 (41.5%)	3 (2.3%)	2 (1.5%)	130

Only one of the isolated head fractures was an open injury following a sports sprain injury.

4) Combined body & neck fractures

This type comprised 54 cases (14.6%), making it the third most common type. Interestingly, all of the combined body & neck fractures were closed injuries. Among Hawkins type III cases, the direction of the fractured-dislocated body was posteromedial to the ankle joint except one posterolateral dislocation (96.5% vs 3.5%).

5) Head fracture combinations

**Fig. 5:**
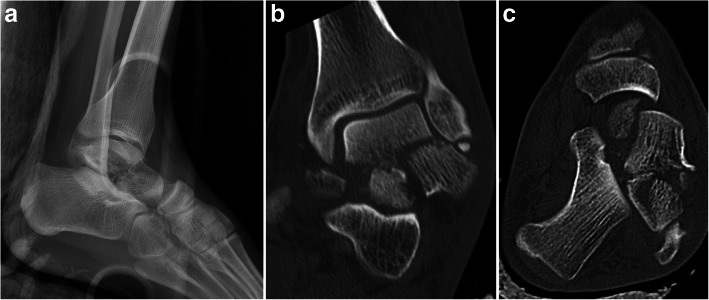
Concomitant head, body & neck fracture of the talus associated with talonavicular subluxation in a young man. Radiograph (**a**), CT sections (**b** & **c**)

There were six cases of head fractures with concomitant fracture of the other parts of the talus: One case with concomitant fracture of body, three cases in association with neck fractures, and two cases of concomitant head, body & neck fractures with talonavicular subluxation (Fig. 5).

### Process fractures

The frequency and demographic data of all kinds of process fractures of the talus are demonstrated in Tables 2 and 5.

1. Isolated lateral process fractures

**Table 5:** Distribution of talar process fractures by age and gender

Age & gender	−18		18–39		40–64		65+		Male/Female ratio	Total
	♂	♀	♂	♀	♂	♀	♂	♀		108
Isolated lateral process	1	4	2	32	2	11	0	1	9.5	53 (49.1%)
Isolated posterior process	2	2	4^a^	15	1	5	0	1	9.5	30 (27.8%)
Isolated medial tubercle	0	1	2	5	1	2	1	0	2	12 (11.1%)
Combined lateral & posterior processes	0	0	6	0	2	0	0	0	–	8 (7.4%)
Medial tubercle combinations	0	0	1	0	3	0	0	1	–	5 (4.6%)

^a^A bilateral isolated posterior process fractures

There were 53 cases of isolated lateral process fractures as the most frequent fractured talar process (49.1%). All were closed injuries except one. Most injured cases were between 18 and 39 years of age.

2) Isolated posterior process fractures

There were 29 cases with 30 closed isolated posterior process fractures of the talus. There was a case of bilateral posterior process fractures after a MVA. He had a comminuted fracture on the right and a simple fracture on the left.

3) Isolated medial tubercle fractures

There were 12 cases with mean age 49.8 ± 14 years. One of them was an open injury.

4) Combined lateral & posterior processes fractures

There were eight men with mean age of 34.3 ± 11.6 years.

5) Medial tubercle fracture combinations

There were four cases of concomitant lateral process of talus fracture and medial tubercle fracture. There was one case of associated posterior process fracture with medial tubercle fracture. The mechanism of injury in all cases was fall. The mean age of patients was 34.3 ± 11.6 years.

### Osteochondral fractures

**Fig. 6:**
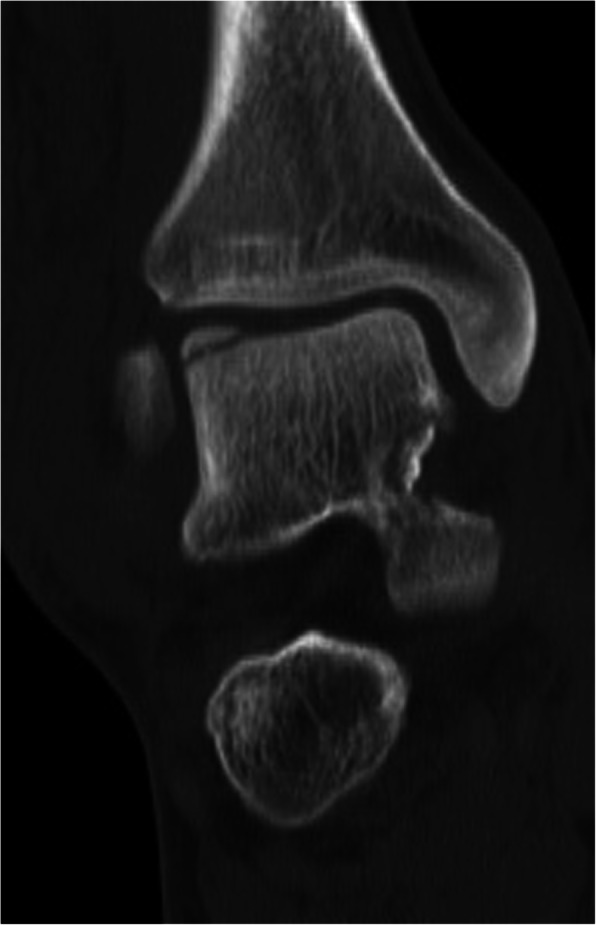
A case of simultaneous lateral dome talus osteochondral fracture and medial body fracture

There were 17 cases (mean age: 22.8 ± 2.2 years, 16 males) of closed osteochondral talar fractures. Five (29.4%) cases had an osteochondral fracture of talar body (two lateral and three medial) and 11 (64.7%) had an osteochondral fracture of dome talus (eight lateral and three medial). There was one case (5.9%) of simultaneous lateral dome talus osteochondral fracture and medial body fracture (Fig. 6).

### Pantalar dislocations

**Fig. 7:**
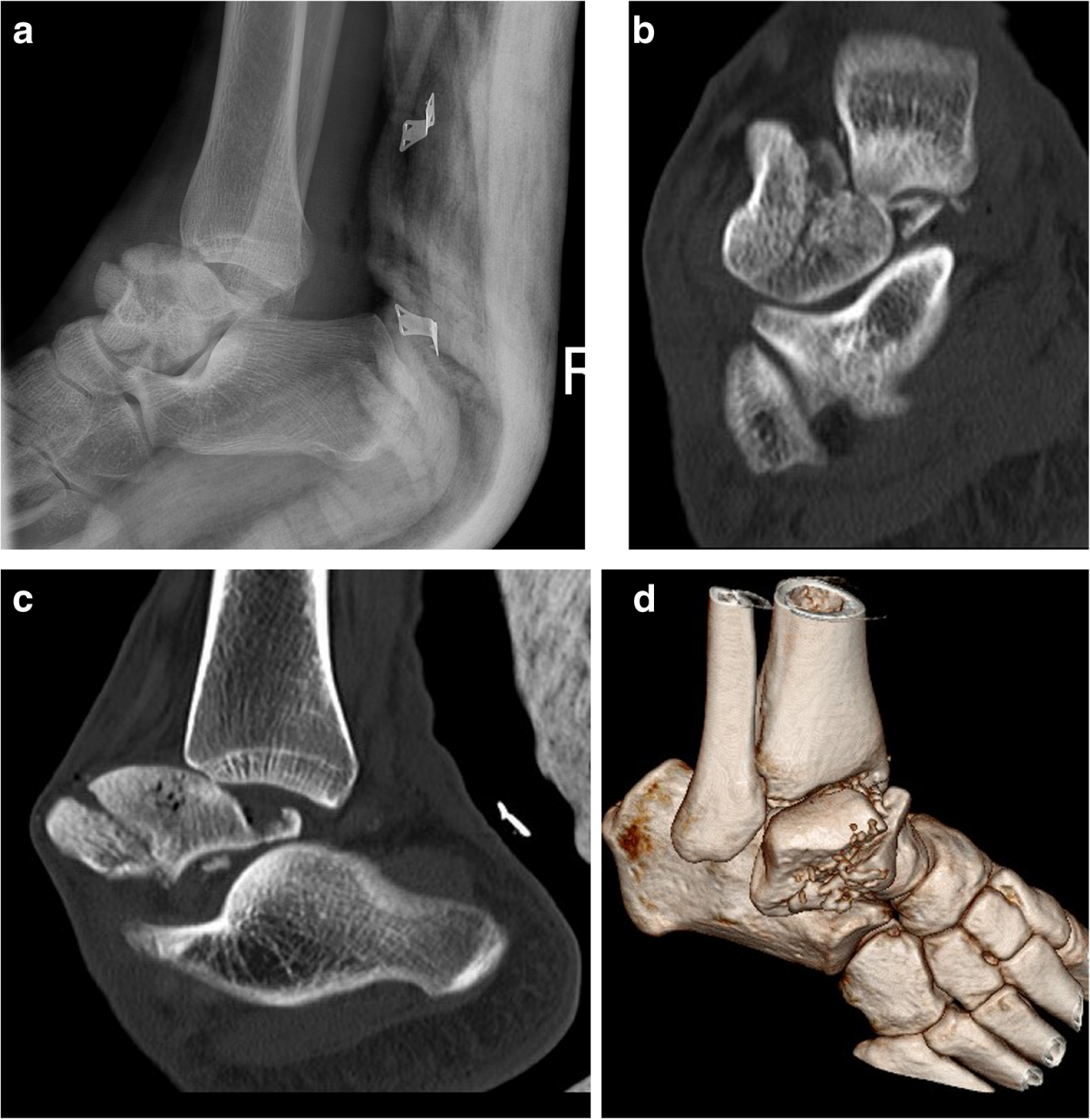
Pantalar fracture dislocation with comminuted fracture of head & neck in a 26-year-old man. Radiograph (**a**), CT sections (**b**-**d**)

**Fig. 8:**
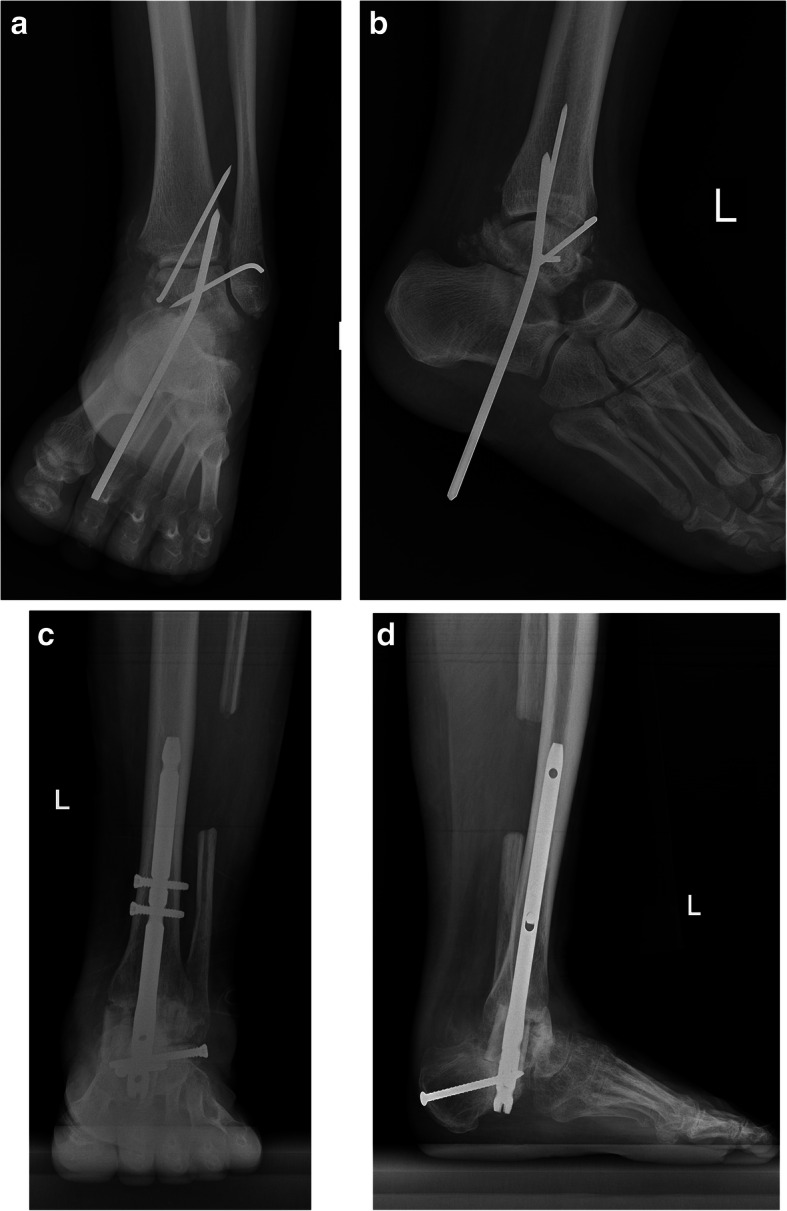
Open pantalar dislocation with lost talar head and medial malleolus (**a** & **b**). Due to infection, after multiple debridement, the talus was resected. In order to prevent limb shortening, the free space between calcaneous and tibia were filled by 3 structural bone graft from fibula and fusion by a hindfoot fusion nail, 7 months after the surgery (**c** & **d**)

There were six cases (mean age: 33.2 ± 12.2 years, 5 males) of pantalar dislocation, half of which were open injuries. The direction of dislocation was anterolateral in all cases, except one with medial direction. Head and neck fracture of talus (Fig. 7), posterior process of talus, tibia plafond, and a lost head of talus (Fig. 8) were the associated fractures.

### Subtalar dislocations

**Fig. 9:**
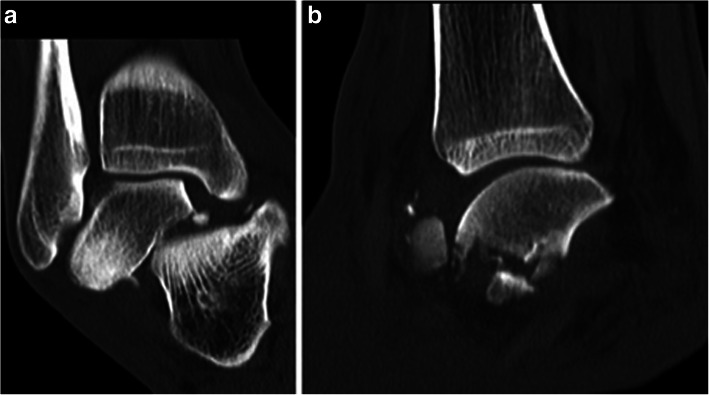
Medial subtalar dislocation associated with talar neck fracture in a 27-year-old man. CT sections (**a** & **b**)

There were three cases of closed medial subtalar dislocation. One occurred in combination with a talar neck fracture and one with a talar body fracture (Fig. 9). All patients were male with a mean age of 35 years (range, 22–43).

### Unspecified fractures

**Fig. 10:**
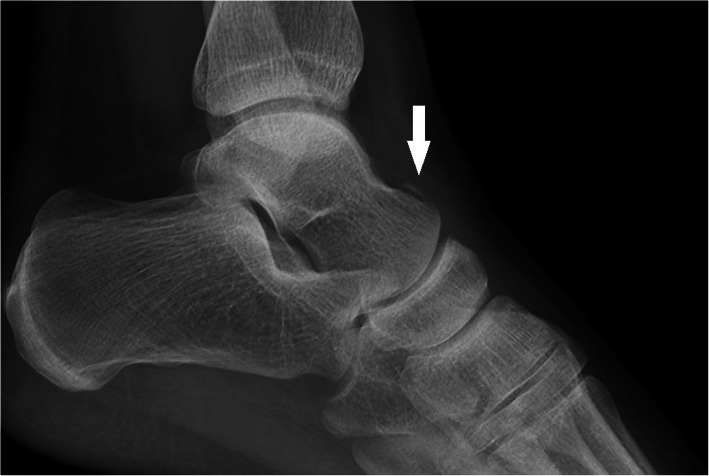
Dorsal neck avulsion fracture

**Fig. 11:**
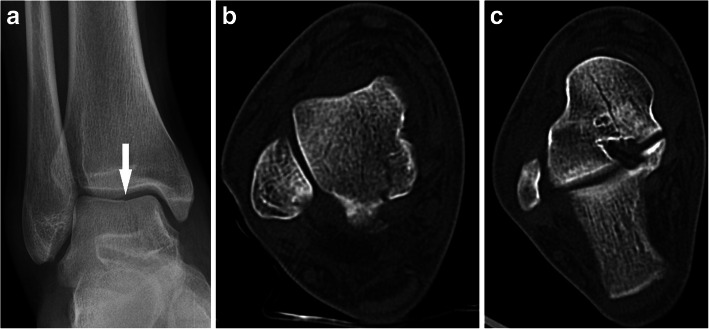
Sagittal nondisplaced fracture of body, neck & head in a 28-year-old man. Radiograph (**a**), CT sections (**b** & **c**)

**Fig. 12:**
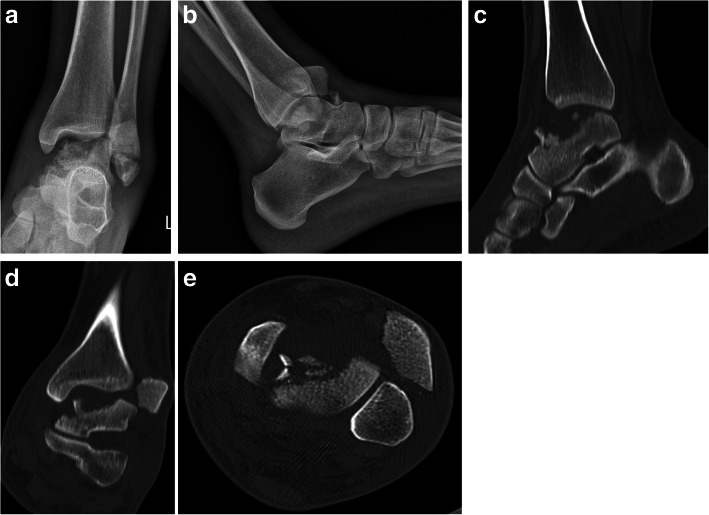
A big displaced partial talar body fracture in association with lateral malleolus fracture in a young woman. Radiographs (**a** & **b**), CT cuts (**c**-**e**)

Ten (2.7%) cases were not categorized in any of the previous groups (Table 6, Figs. 10, 11 and 12).

**Table 6:** Unspecified fractures of the talus

	Number of cases	Age mean ± SD	mechanism of injury
Dorsal neck avulsion fracture	4	28.3 ± 5.7	Fall: 33.3%, MVA: 66.7%
Medial neck avulsion fracture	1	55	Fall
Sagittal fracture of body, neck & head	1	28	Unknown
Pathologic neck fracture	1	30	Fall
Partial body fracture, displaced in anterior ankle	1	23	Fall
Talocalcaneal coalition fracture	2	22.0 ± 1.4	Fall

*MVA* Motor Vehicle Accident

### Bilateral fractures

Three patients presented with bilateral talar fracture (0.8%).

- A 32-year-old man with a right displaced talar body and left combined body & neck fracture Hawkins type IIA after a fall.
- A 30-year-old woman with bilateral neck fractures (Right: Hawkins type IIA; Left: Hawkins type I), after a car turn-over crash.
- A 33-year-old man with bilateral posterior process fractures following a car-to-car accident.

## Discussion

Fractures and/or dislocations of the talus are rare injuries without any large case-based epidemiologic study to clarify the characteristics of different injury patterns. Based on our study, the most injured patients were young males between 18 to 45 years. It is notable that the frequency of talar injuries decreases with increasing age with two peaks (20–25 years and 30–35 years) and a significant reduction in the elderly who are more than 65 years old. These findings are consistent with those of the previous studies [1, 13]. The male to female ratio of 6.3 which was reported in the present study is more than 4.8 in two previous studies from Brazil [1, 9]. This may be due to the fact that the most difficult or risky jobs are performed by men.

Notably, we had only 7.0% open talar injuries which are far less than 31.4% reported in the previous studies on talus fractures [1] and in comparison, to the rate of open fractures among all foot and ankle fractures [16]. Interestingly, none of our type III talar neck fractures were open, while others stated that 50% of their fractures were open injuries [24].

The most frequent mechanism of injury was MVA in our study. This is similar to the previous studies [9, 13, 15, 25]. Conversely, falls predominated as the mechanism of injury in a study by Mugnol. et al. from Brazil [1]. This may be due to the variation of potential social and occupational risks in different countries that is important for employment protection legislation. About half of the patients in our study injured by the mechanism of MVA were motorcyclists. This clarifies the importance of strict legislation by health policymakers and governments for motorcyclists.

Talar injuries are usually the result of high-energy impacts and are frequently seen in poly-traumatized patients. Previous studies reported the prevalence of associated injuries about 47 to 89% [1, 9, 13]. In our study, about 62% of the patients had at least one associated injury, mostly ipsilateral. The most common accompanying injuries were medial malleolus (34.5%), lateral malleolus (fibula) (28.8%) and calcaneus (22.7%). Our results are the same as the data previously gathered [13], except for one that described calcaneus fracture as the most frequent associated injury [1]. It is deduced that patients with foot and ankle injuries resulting from MVAs need a thorough evaluation because they are more likely to suffer from fractures than other types of injuries, such as ankle sprains. Furthermore, it is important to evaluate the other foot and ankle bones, especially the malleoli and calcaneus, when encountering a patient with talar injuries and vice versa.

According to our results, fractures of body, head, and neck of the talus (61.1%) were twice more frequent than talar process fractures (29.2%). This is contrary to what was reported by Mugnol. et al. [1]. On the other hand, Sakaki et al. [9] reported 87% neck and body fracture of the talus with the highest incidence for Hawkins type II with no type I neck fracture. In our study, isolated talar body fracture was the most prevalent type, accounting for 21.9% of cases, followed by isolated neck fracture (19.2%). Hawkins type II was the most common type followed by III, I, and IV in all cases with talar neck fractures.

Remarkably, pantalar dislocations (1.6%) were more frequent than the subtalar dislocation (0.8%) in our study. Pantalar dislocation, especially closed types, are very rare [26]. We think that this may be a bias of our study because the most severely injured cases are usually referred and admitted to our level I trauma center. Subtalar dislocations, especially simple ones without associated fractures, may be reduced and treated in local or private hospitals.

Our research is the biggest population-based study on all types of talar injuries in the literature. Importantly, all talar injuries referred to our centers are routinely requested to take an ankle CT scan which ensures precise typing of talar injuries and possible associated injuries that may have a considerable role in the management of foot and ankle fractures. The other strong point was reviewing all CT scans by a single orthopedic foot and ankle surgeon. We included all talar injuries like peri-talar dislocations, which was not described in the previous studies [1, 9, 13, 25].

One of the limitations in our study was the lack of availability of injury mechanisms of all patients; however, we tried to get them as much as possible by phone contacts.

## Conclusions

The population that is most affected by this injury are active young men who are involved in motor vehicle accidents, especially motorcycle crashes, with fracture of body and/or neck of talus being the most common type of talar injury in these patients. Further studies on each type of talar injuries in different societies should be carried out to illuminate different patterns and characteristics of these injuries.

## Data Availability

The datasets used and/or analyzed during the current study are available from the corresponding author on reasonable request.

## Abbreviations

MVA: Motor Vehicle Accident
CT: Computed Tomography
